# Liver Transplantation and Gut Microbiota Profiling in a Child Colonized by a Multi-Drug Resistant *Klebsiella pneumoniae*: A New Approach to Move from Antibiotic to “Eubiotic” Control of Microbial Resistance

**DOI:** 10.3390/ijms19051280

**Published:** 2018-04-25

**Authors:** Federica Del Chierico, Sabrina Cardile, Andrea Pietrobattista, Daniela Liccardo, Alessandra Russo, Manila Candusso, Maria Sole Basso, Chiara Grimaldi, Laura Pansani, Paola Bernaschi, Giuliano Torre, Lorenza Putignani

**Affiliations:** 1 Unit of Human Microbiome, Bambino Gesù Children’s Hospital, IRCCS, Viale San Paolo 15, 00146 Rome, Italy; federica.delchierico@opbg.net (F.D.C.); alessandra.russo@opbg.net (A.R.); 2 Unit of Gastroenterology, Hepatology and Nutrition, Bambino Gesù Children’s Hospital, IRCCS, Piazza Sant’ Onofrio 4, 00165 Rome, Italy; sabrina.cardile@opbg.net (S.C.); andrea.pietrobattista@opbg.net (A.P.); daniela.liccardo@opbg.net (D.L.); manila.candusso@opbg.net (M.C.); msole.basso@opbg.net (M.S.B.); giuliano.torre@opbg.net (G.T.); 3 Unit of Hepatobiliary Surgery and Abdominal Transplant, Bambino Gesù Children’s Hospital, IRCCS, Piazza Sant’Onofrio 4, 00165 Rome, Italy; chiara.grimaldi@opbg.net; 4 Unit of Microbiology, Bambino Gesù Children’s Hospital, IRCCS, Piazza Sant’ Onofrio 4, 00165 Rome, Italy; laura.pansani@opbg.net (L.P.); paola.bernaschi@opbg.net (P.B.); 5 Unit of Parasitology, Bambino Gesù Children’s Hospital, IRCCS, Piazza Sant’ Onofrio 4, 00165 Rome, Italy

**Keywords:** carbapenem resistant-*Klebsiella pneumoniae*, gut microbiota, liver diseases, liver transplantion, microorganisms multi-drug resistant, pediatrics

## Abstract

The increase of microorganisms multi-drug resistant (MDR) to antibiotics (ATBs) is becoming a global emergency, especially in frail subjects. In chronic liver disease (LD) with indications for liver transplantation (LT), MDR colonization can significantly affect the LT outcome. However, no clear guidelines for microbial management are available. A novel approach toward MDR-colonized patients undergoing LT was developed at our Center refraining from ATBs use during the transplant waiting list, and use of an intensive perioperative prophylaxis cycle. This study aimed to couple clinical evaluation with monitoring of gut microbiota in a pediatric LD patient colonized with MDR *Klebsiella pneumoniae* (KP) who underwent LT. No peri-transplant complications were reported, and a decontamination from the MDR bacteria occurred during follow-up. Significant changes in gut microbiota, especially during ATB treatment, were reported by microbiota profiling. Patterns of *Klebsiella* predominance and microbiota diversity revealed opposite temporal trends, with *Klebsiella* ecological microbiota niches linked to ATB-driven selection. Our infection control program appeared to control complications following LT in an MDR-KP-colonized patient. The perioperative ATB regimen, acting as LT prophylaxis, triggered MDR-KP overgrowth and gut dysbiosis, but buffered infectious processes. Mechanisms modulating the gut ecosystem should be taken into account in MDR colonization clinical management.

## 1. Background

Resistance to antibiotics is becoming a global emergency, especially in industrialized countries; hence, reduction of such resistance is a current public health challenge. In fact, according to the Centers for Disease Control and Prevention (CDC), antibiotic-resistant microbes cause more than 2 million illnesses and at least 23,000 deaths each year in the US (http://www.cdc.gov/drugresistance/). Of note, during the last decade, carbapenem resistant-*Klebsiella pneumoniae* (CR-KP) has been spreading worldwide and becoming a serious healthcare issue in a growing number of countries, including Italy [1]. Appropriate management of patients colonized with multi-drug resistant (MDR) bacteria is crucial in frail patients, particularly pediatric, elderly, hospitalized, immunosuppressed and chronically ill patients [2]. Limiting the use of empirical antibiotic (ATB) therapy, especially in patients hospitalized in intensive care units, is an optimal, proven strategy for reducing infection/colonization by MDR bacteria [3]. In children, a recent multicenter study detected MDR among 33.6% of Gram-negative bloodstream infections, most frequently among infections with *Escherichia coli*, *Enterobacter* spp. and *Pseudomonas* spp. [4]. Typing strategies of CR-KP are based on pulsed field gel electrophoresis (PFGE), repetitive-sequence-based PCR (rep-PCR) and matrix-assisted laser desorption/ionization time-of-flight mass spectrometry (MALDI-TOF MS)-based proteomic phenotyping [5,6,7]. 

In patients with chronic liver disease (LD) with indications for liver transplantation (LT), MDR colonization can significantly affect the transplant outcome: hence, patients-tailored diagnostic and therapeutic approaches are needed [8].

LT recipients, especially children, are reported to be at especially high risk of acquiring and dying from CR-KP infections [9]. Despite strong recommendations for the prevention of CR-KP transmission in health care facilities, universal fecal screening of asymptomatic LT candidates and/or recipients is not the standard of care and remains an uncommon practice [10,11]. Further, there is no consensus about the management of CR-KP colonized patients during waiting list and undergoing liver transplant, especially if infants or children. Currently, therapeutic strategies include liver transplant deferring, pre-transplant selective intestinal decontamination and peri-operative ATB prophylaxis. Moreover, the limited data about clinical management options and outcome prevents definite conclusions about how to manage LT candidates with persistent CR-KP carriage [12]. 

The gut is a large reservoir of MDR bacteria, including naturally and acquired resistant phenotypes [13]. The density of MDR microorganisms can reach high concentrations in the gastrointestinal (GI) tract; therefore, a colonized patient presents an epidemiological threat to other hospitalized individuals and to himself [14]. The GI MDR bacteria are high risk factors for life-threatening systemic infections, especially in the presence of immunosuppression (IS) and GI damage, which drives MDR translocation into bloodstream [15]. However, eubiotic gut microbiota guarantees protection from gut colonization by MDR organisms, through colonization resistance [14]. Indeed, commensal bacteria and molecules they release stimulate GI mucosal immunity and increase resistance against colonization by *Clostridium difficile*, Enterobacteriaceae (e.g., CR-KP, ESBL-producing *E. coli*), and vancomicyn-resistant Enterococci (VRE) [16]. One of the major causes of gut dysbiosis is administration of broad-spectrum ATBs, which increases susceptibility to such colonization by inducing metabolic imbalance of the gut ecosystem [17]. This outcome is caused by bactericidal effects, but also by alterations in gut microbiota/host enterocyte interactions, such as declining IL-17 and T cells stimulation, causing further imbalance [18]. Therefore, ATB treatment that kills commensal microorganisms decreases microbiota-mediated innate immune defenses and so enables residual ATB-resistant species to proliferate and dominate mucosal surfaces [16].

## 2. Case Presentation

A 3-month-old Caucasian female with a late diagnosis of biliary atresia, not subjected to Kasai portoenterostomy, developed advanced LD with portal hypertension, splenomegaly, ascites and mild coagulopathy; hence LT was considered the only reasonable option. As per our protocol, the patient and her parents underwent wide infectious disease screening exams. Although parents’ exams were negative, the patient’s rectal swab was CR-KP positive, with a microbial sensitivity test (MST) compatible with an MDR pattern. All other sample (i.e., blood, urine, etc.) cultures were negative. Because symptoms and signs of invasive infection were absent, intestinal colonization with carriage of CR-KP was diagnosed. Regular monitoring using culture- and metagenomics (MG)-based gut microbiota profiling was performed. 

Written informed consent was obtained from the patient’s parents for inclusion in the study and for publication of this manuscript. The study was conducted in accordance with the Declaration of Helsinki, and the protocol was approved by the UniCatt and Bambino Gesù Children’s Hospital Ethics Committees (Protocol Number 9759/15, 26 March 2015).

Previously to her first evaluation at our Center, this girl was admitted to a local hospital at 1 month old of age for a urinary tract infection caused by KP and treated with oral ciprofloxacin. No other prior hospital admissions or ATB therapy were recorded in her clinical record. Once KP colonization was ascertained, ATB treatments were avoided, unless strongly indicated, during the transplant waiting list. She underwent living donor LT from father when she was 11 months old and weighed 8.7 kg. The protocol of perioperative prophylaxis, usually lacking ATB treatment prior intervention, was modified accordingly to MST results. Hence, she received 24 h of oral colimicyn as “direct” decontamination, followed by amikacin/colimicyn and amphotericin B intravenous regimen after surgery.

The standard immunosuppressive regime with basiliximab, steroids bolus plus oral tacrolimus was started. No surgical complications were reported. One week after LT, all cultures were negative except stool cultures, which were still CR-KP positive. On the 8th postoperative day, fever and elevation of inflammatory markers were observed. Peritoneal culture grew both CR-KP and *Enterococcus faecalis* sensitive to vancomicyn. Therefore, the ATB regimen was immediately modified: tigecycline, vancomicyn and metronidazole were begun and colimicyn administered only by peritoneal irrigation. 

The girl improved clinically: fever resolved after 48 h, inflammatory markers declined promptly and peritoneal culture were negative within 4 days. All other cultures, especially blood cultures, remained negative. ATB treatment was tapered until suspension within 3 weeks. 

Four weeks after transplant, she was discharged in good clinical condition, gaining weight, with a normal liver graft regardless of persistent positive CR-KP stool cultures. At follow up, no surgical or immunological graft complications were observed and no hospital admission was required. Except for *Pneumocystis jirovecii pneumonia* prophylaxis, she did not receive any further ATB treatment. 

Culture- and MG-based gut microbiota monitoring was continued after discharge to investigate ongoing modifications. CR-KP intestinal carriage persisted during the first 5 months after LT; however, on day 164 this test became negative and remained negative through the last follow up visit, 20 months after LT. 

The MG-based gut microbiota profiling was performed on 16 consecutive fecal samples. Genomic DNA was isolated by QIAamp DNA Stool Mini Kit (Qiagen, Hilden, Germany). A region of 520 base pairs (V1–V3) of the 16S ribosomal RNA (rRNA) locus was amplified using barcoded primers (Forward 5′-GAGTTTGATCNTGGCTCAG-3′, Reverse 5′-GTNTTACNGCGGCKGCTG-3′). The next pyrosequencing step was performed on a 454-Junior Genome Sequencer (Roche 454 Life Sciences, Branford, CT, USA) [19].

Obtained sequences were analyzed by using QIIME 1.8.0 software [20]. Sequences were denoised [21] and chimera-checked (http://qiime.org/scripts/identify_chimeric_seqs.html). The operational taxonomic units (OTUs) defined by a 97% of similarity were picked, aligned by PyNAST [20], and clustered by UCLUST [22]. Greengenes database (v 13.8) was employed for OTUs matching. The Shannon index (SI) were calculated using QIIME software (alpha_rarefaction.py script) [20]. Statistical analysis was performed using SPSS 20 software (IBM SPSS Statistics for Windows, Version 20.0., IBM Corp., Armonk, NY, USA).

A total of 161,485 sequencing reads were obtained from all samples, with a mean of 3938 sequence for sample.

**Figure 1:**
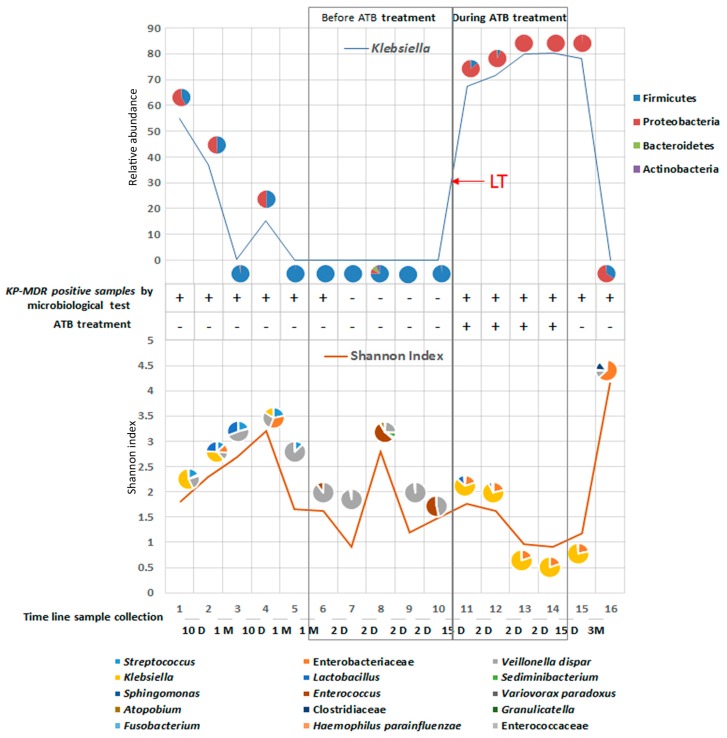
A combined approach of metagenomics-based gut microbiota profiling and culture-based MDR KP tracing pre- and post-transplantation. The upper graph shows relative abundance of *Klebsiella* during the microbiota profiling sample timeline, within the context of phyla distribution at each sampling point. The lower graph shows Shannon biodiversity index and species/family distribution during the timeline. The central table reports MDR *K. pneumoniae* isolations by culture-based microbiological methods and the antibiotic (ATB) treatment. The bottom dashed line indicates the time between each sample. D, days; M, Months. The arrow indicated the date of the liver transplant (LT).

At first, the microbiota was comprised of 50% Firmicutes and 50% Proteobacteria, and the percentage of *Klebsiella* abundance decreased from 55% to 37% in the first two samples (Figure 1). 

**Figure 2:**
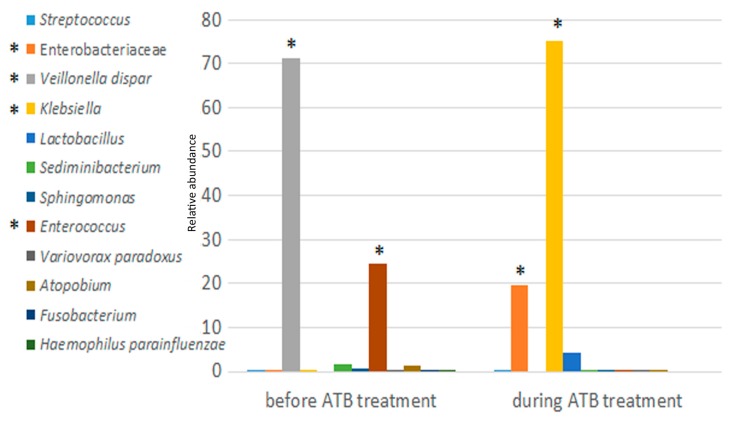
Bar chart comparing operational taxonomic unit (OTU) relative abundances before and during ATB treatment. OTUs at species/family levels were compared using the Mann-Whitney test. Samples were grouped and averaged in “before” and “during ATB treatment” groups of samples. Taxonomic composition is shown at species/family levels. Each column color represents the relative abundance for each OTU. Statistically significant differences (*p* < 0.05) are indicated by an asterisk.

After one month, *Klebsiella* declined to 0.4% and Firmicutes increased to 98% of the microbiota. In these samples, the SI increased from 1.5 to 3; as *Klebsiella* distribution decreased until undetected, *Veillonella parvula*, *Streptococcus*, *Lactobacillus* and Enterobacteriaceae increased. During the following month, *Klebsiella* levels increased, reaching 15% with a fluctuating pattern. At this point, the microbiota was comprised of 50% Firmicutes and 50% Proteobacteria. In the last samples collected before LT, except for the eighth sample, Firmicutes (i.e., *V. parvula*) populated the entire microbiota, while *Klebsiella* was undetected (Figure 1). Then, the child underwent LT and ATBs started. Twelve days after LT, *Klebsiella* abundance reached 66%, and the microbiota was comprised of 85% of Proteobacteria (i.e., *Klebsiella* and Enterobacteriaceae) and 14% of Firmicutes (i.e., *Lactobacillus*). During the following days, Proteobacteria constituted 99.9% (i.e., 80.2% *Klebsiella* and 19.7% Enterobacteriaceae) of the microbiota and the SI reached the absolute minimum value of the curve. Fifteen days after ATB treatment was stopped, the gut microbiota was comprised of 99.3% Proteobacteria (i.e., 78.2% *Klebsiella* and 20.9% Enterobacteriaceae). After three months, in the last sample, *Klebsiella* was replaced by an eubiotic microbiota, and the microbiota was comprised of Enterobacteriaceae, *V. dispar*, Clostridiaceae, *Granulicatella*, *Fusobacterium*, *Haemophilus parainfluenzae*, Enterococcaceae and *Streptococcus.* This microbial diversity was reflected by the maximum SI value (Figure 1). The OTUs at species/family levels of two categories of samples, “before ATB treatment” versus “during ATB treatment”, were compared using the Mann-Whitney test; significant differences were found for *Klebsiella*, Enterobacteriaceae, *V. parvula* and *Enterococcus*, confirming their high fluctuation (Figure 2). 

**Figure 3:**
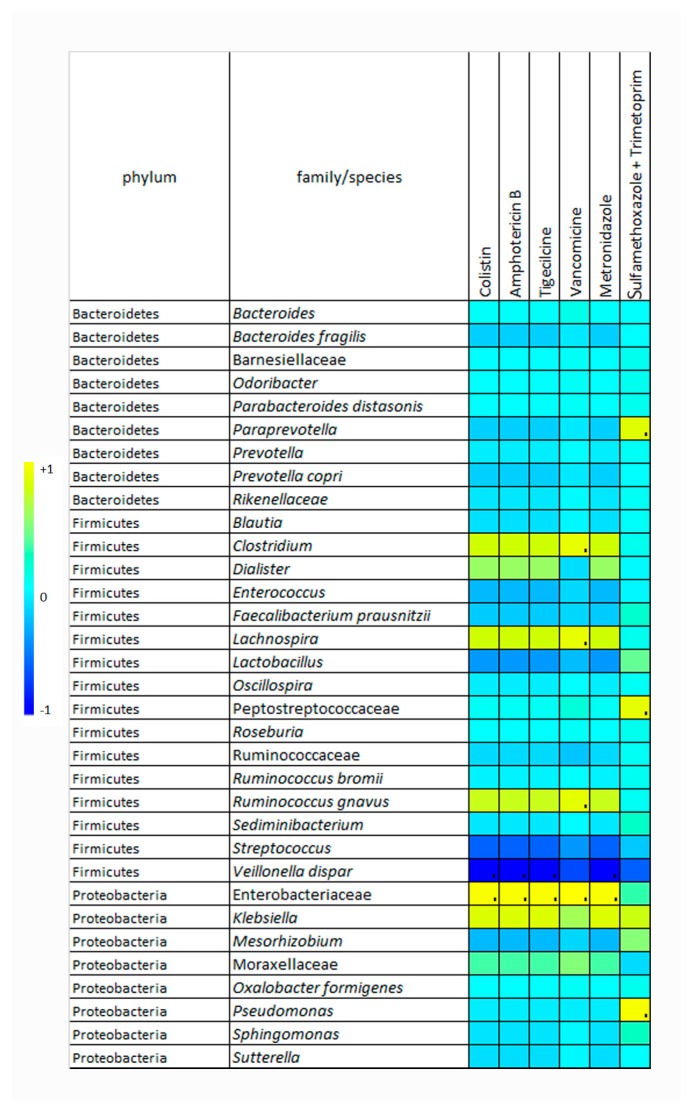
Heat-map depicting correlations between operational taxonomic unit (OTU) abundance and antibiotic administration. The map displays the Spearman correlation coefficients (represented by colored squares) for each OTU. The color of each square indicates the direction of the correlation (i.e., yellow positive and blue negative correlation) and the color intensity of each square indicates the correlation value; greater intensity indicates stronger correlation.

The correlations between OTU abundance and ABT administration were evaluated by the Spearman’s test considering the whole dataset. *Streptococcus* and *V. dispar* were negatively correlated with ATBs, and Enterobacteriaceae, *Klebsiella*, *Ruminococcus gnavus*, *Lachnospira*, and *Clostridium* were strongly correlated with ATBs (Figure 3).

## 3. Review and Discussion 

Recently, the risk of disseminated infection for CR-KP colonized patients has increased for abdominal surgery, intestinal and biliary anastomoses, transplantation-linked IS and chronic illnesses. However, the incidence of nosocomial bloodstream infections appears similar in transplanted patients and the general population, although antimicrobial resistance, especially of Gram-negative bacteria, is higher in transplanted recipients [2]. Giannella M. et al. [23] investigated the time course of CR-KP infections and colonization after LT and showed that CR-KP induced infections were preceded by colonization in all patients, and that most patients became colonized by CR-KP during hospitalization. In fact, recent studies have shown through genomic evidence that the gut microbiota is a substantial source of hospital- acquired KP infections [24]. Thus far, the medical literature does not provide definitive recommendations for prophylactic management of antimicrobial resistance in transplant recipients, de facto broadening ATB resistance risk and narrowing the range of ATB molecules that can be administered in the post-transplant period. 

However, the high rate of complications leads to an increased risk of post-transplant mortality due to disseminated infection. Indeed, resistance to carbapenems by Enterobacteriaceae is increasingly concerning because *E. coli* and *Klebsiella* are a growing cause of nosocomial and community acquired infections [25,26]. 

Based on this illustrative case, we have described the changes in the gut microbiota composition related to ATB administered for LT by plotting abundance of *Klebsiella* and SI values over the timeline (Figure 1). 

At phylum and species levels, the microbiota profile changed greatly over time. Particularly, *Klebsiella* abundance was negatively correlated with microbial diversity as measured by the SI, showing the ability of these microorganisms to overgrow, under ATB selective pressure, and fill the entire ecological niche of microbiota, after suppression of other ATB sensitive species. Exposure of gut microbiota to ATBs is known to reduce microbial susceptibility to drugs and can induce development of MDR microorganisms [27,28]. Of note, ATBs can impair the ecology of commensal species, leading to a reduction of intrinsic colonization resistance and contributing to the transmission of ATB resistance mechanisms, such as lateral transfer of resistance genes [29,30].

Consequently, once colonization by MDR-KP was found in this patient, we avoided ATB administration, except for one episode of acute severe cholangitis treated with ciprofloxacin, and frequent hospital admissions obtaining KP negative culture- and MG-based results. However, at the time of transplant, we were no longer aware of the gut microbiota results, nor did reported studies support the role of gut microbiota in MDR-KP colonized patients. Therefore, we based our ATB treatment of MDR-KP on MST results, without considering the ecological implications for other microbiota species or dysbiosis patterns.

Previously, gut microbiota was thought to be relatively resilient to ATB effects, and that, after short-term modification, returns to their pre-treatment composition [31]. However, ATB therapies are now known to have long-term effects on intestinal bacteria, which can increase the risk of ATB resistance and transfer resistance to pathogens. Comparison of the GI community structure of three healthy subjects, before and after ciprofloxacin treatment, revealed that this ATB led to an immediate reduction of species diversity. Furthermore, although the recovery of microbiota composition began to appear 1-week post-treatment, complete recovery of eubiosis was not observed until 6–10 months after ATB cessation [32,33].

Numerous strategies have attempted to control ATB resistance and overgrowth of MDR bacteria during ATB treatment, especially in immunosuppressed patients. These prevention approaches include oral digestive decontamination to prevent nosocomial infections and ATB administration (i.e., selective digestive decontamination, SDD) consisting of non-absorbable ATBs (e.g., aminoglycosides and polymyxin E) applied to the oropharyngeal cavity and administered into the stomach, for three days. Indeed, this approach has been reported to decrease early-onset infections, secondary endogenous colonization, and gut endotoxin content [34]. 

Based on this evidence and our experience, the day before surgery we started oral decontamination with colimicyn, aiming at increasing ATB coverage during the most critical period of the transplant itself. This approach, adopted only in patients with MDR colonization, is possible, although still not standardized, but limited to patients undergoing living donor LT because for recipients of cadaveric transplants decontamination can be started only after a compatible organ is confirmed. 

In our case, at the time of transplantation, the limited and MST-recommended ATB regimen controlled systemic dissemination of MDR-KP and provided synergistic ATBs, instead of standard ATBs protocol based on ampicillin and second-generation cephalosporin. However, we did not consider the ecology of the microbiota and the profound negative effect that prolonged ATB treatment has on other microorganisms, causing dysbiosis after LT.

Recent evidence suggests that administration of prebiotics and probiotics is the most effective strategy to maintain or restore the beneficial commensal populations of gut microbiota during or after ATB administration [35]. Although prebiotics cannot restore bacterial communities that are unbalanced due to ATB therapy, they may be used in synergistic combinations with probiotics to increase indigenous commensal species and to produce health-promoting metabolic products (i.e., short chain fatty acids), which can prevent the overgrowth of endogenous or external pathogenic bacteria. Fecal microbiome transplantation (FMT), that is producing excellent results in *Costridium difficile* relapses, might be an innovative approach against MDR colonization. FMT consists of administering fecal microbiota from a normal individual into the gut of an affected patient with the goal of achieving colonization with a well-balanced community of microorganisms [36,37,38,39]. 

Soon, monitoring of microbiota status using stool culture and MG coupled profiling before and after transplantation will greatly contribute to decisions about perioperative ATB prophylaxis and treatment and also support more “physiological” interventions for eubiosis recovery, such as use of pre- and probiotics and/or FMT.

The present report may provide groundbreaking results that suggest new approaches for the management of transplant recipients infected or colonized with MDR-KP. It may stimulate discussion of targeted intervention strategies leading to alternative control of bacterial translocation and related systemic infections, exploiting eubiotic management of the gut microbiota, including MDR microbes, rather than endorsing greater ATB use.

## 4. Conclusions

Infections with CR-KP are associated with high morbidity and mortality, particularly among vulnerable patient populations, such as those undergoing solid organ transplantation. Our strategy to optimally manage the discussed case of an MDR colonized patient awaiting and ongoing LT suggests attention to the following strategies: (i) reduction of ATB pressure and ad hoc usage of sensitive molecules; (ii) abstention, during the LT waiting list, of ATB therapies, especially those MDR-targeted; and (iii) limited duration of intensive cycles of perioperative prophylaxis, which have proven effectiveness in reducing peri-transplant mortality. Moreover, the swift reintegration post-transplant of the child within the family with avoidance of a prolonged hospitalization allowed the spontaneous and slow decontamination of the microbe, despite a steady IS therapy. 

Emerging data from microbiota profiling may provide new indications on the use of eubiotic strategies not only addressing the microbes’ eradication (e.g., ATB use), but also the microbes’ control within its ecosystem. Lack of microbial biodiversity should be considered the best indication of development of MDR microbes and suggests the need to prevent unremitting or cyclic ATB-induced dysbiosis through thoughtful complementary approaches such as probiotic administration and FMT into clinical protocols. 

## Abbreviations

ATBs	Antibiotics
CDC	Centers for disease control and prevention
CR	Carbapenem resistant
FMT	Fecal microbiome transplantation
GI	Gastrointestinal
KP	*Klebsiella pneumoniae*
LD	Liver disease
LT	Liver transplantation
MALDI-TOF MS	Matrix-assisted laser desorption/ionization time-of-flight mass spectrometry
MDR	Multi-drug resistant
MG	Metagenomics
MST	Microbial sensitivity test
OUT	Operational taxonomic unit
PFGE	Pulsed field gel electrophoresis
rep-PCR	Repetitive-sequence based PCR
SDD	Selective digestive decontamination
SI	Shannon index
VRE	Vancomicyn-resistant Enterococci
